# A qualitative study of providers’ perceptions of parental feeding practices of infants and toddlers to prevent childhood obesity

**DOI:** 10.1186/s12889-021-11305-7

**Published:** 2021-06-30

**Authors:** Rebecca L. Heller, Jesse D. Chiero, Nancy Trout, Amy R. Mobley

**Affiliations:** 1 Department of Nutritional Sciences, University of Connecticut, Storrs, CT USA; 2 Rhythm Pharmaceuticals, Boston, MA USA; 3 Division of Primary Care, Connecticut Children’s Medical Center, Department of Pediatrics, University of Connecticut School of Medicine, Hartford, CT USA; 4 Department of Nutritional Sciences, Storrs, CT USA; 5 Department of Health Education and Behavior, University of Florida, PO Box 118210, Gainesville, FL 32611-8210 USA

**Keywords:** Parental feeding, Childhood obesity, Healthcare provider, Infants

## Abstract

**Background:**

With a recent focus on establishing US Dietary Guidance for children ages 0 to 2 years old, the objective of this qualitative study was to determine misconceptions and barriers that prevent parents from implementing early childhood feeding and obesity prevention practices as reported by healthcare, community-based, and education providers.

**Methods:**

Trained researchers conducted one-on-one qualitative phone interviews, using a semi-structured script, with early childhood health and education providers working with families of young children. Interviews were audiotaped, transcribed verbatim, and analyzed using the classic analysis approach. Transcripts were coded by researchers and analyzed for themes.

**Results:**

Providers (*n* = 21) reported commonly observed obesogenic practices including overfeeding tendencies, early initiation of solids or less optimal feeding practices, lack of autonomy and self-regulation by child, and suboptimal dietary patterns. Sources of parental misconceptions about feeding were often related to cultural, familial, and media influences, or lack of knowledge about optimal feeding practices for infants or toddlers.

**Conclusions:**

Providers indicated a need for engaging and consistent child feeding and obesity prevention education materials appropriate for diverse cultural and literacy levels of parents, with detailed information on transitioning to solid foods. Early education and community-based providers reported limited access to evidence-based educational materials more so than healthcare providers. It is an opportune time to develop reputable and evidence-based child feeding guidance that is readily available and accessible for parents of infants and toddlers to prevent early childhood obesity.

## Introduction

Childhood obesity is a public health concern with both short- and long-term health consequences [1, 2]. Approximately one in every 10 children from age birth to 2 years old is at a high weight-for-length percentile [3, 4]. Overweight and obesity in childhood increases the risk of adult obesity and weight-related health conditions indicating a need for early behavioral interventions [1].

Rising childhood obesity rates have been linked to increasing energy balance secondary to caloric intake and age-inappropriate dietary patterns [1, 5]. Children’s dietary behaviors are first formed during the critical ages of birth to 2 years when children are introduced to new foods and transition to an adult diet [6]. Early exposure to new and healthful foods, particularly through repeated exposure and feeding techniques, can help improve dietary behaviors [7–9]. Parents and caregivers serve as the primary direct influence on children’s intake through the types of foods they introduce and the methods they use to do so [9–11]. Specifically, parental feeding styles and practices have been associated with children’s dietary intake and weight status [12–14].

Previous studies have also identified healthcare and education providers as having significant influences on child feeding practices by way of parents and caregivers, with resulting positive and negative outcomes [15–18]. Essential resources for child feeding information are often disseminated by healthcare, community-based, and education providers to parents during the first few years of a child’s life. While health and education providers have an important role in educating parents about early childhood feeding and obesity prevention, several professional agencies that provide child health information sometimes promote conflicting early child feeding information and guidelines [19–21]. For instance, some agencies recommend the introduction of complementary foods as soon as 4 months while others recommend waiting until 6 months of age. Further, some agencies recommend introducing cow’s milk before 12 months of age while others recommend waiting until at least 12 months of age. These conflicting messages may further contribute to inadequate or inappropriate feeding practices through increased confusion among parents and caregivers about proper timing and techniques to implement in early child feeding practices [22]. Despite the evident impact on dietary behaviors and health outcomes, there is limited research-based educational outreach currently targeting child-feeding practices for birth to 2-year-olds [22, 23]. To address the growing issue of early childhood obesity, it is essential to provide parents and caregivers with evidence-based child feeding messages and to understand what obesogenic practices are being observed, their origin, and how providers address feeding issues.

For the first time in history, the US Dietary Guidelines for Americans will include guidance on nutrition needs of children ages 0 to 2 years old [24]. Healthcare, community-based, and education providers can have significant influences on parental child feeding practices [15, 16, 18, 25] and serve as key health communicators of timely and updated dietary guidance for infants and toddlers. The purpose of this qualitative study was to determine misconceptions and barriers that prevent parents from implementing early childhood feeding and obesity prevention practices as reported by healthcare, community-based, and education providers.

## Methods

The study was approved by the University of Connecticut (UConn) Institutional Review Board (IRB) for Human Subjects, Human Research Protection Program, Protocol #H16–029 and conducted in accordance with the Declaration of Helsinki. Written informed consent was waived with approval of the UConn IRB because interviews were conducted by phone. Prior to the interview, each participant reviewed an information sheet and provided verbal informed consent.

### Participants

As part of a larger project by the research team to develop childhood obesity prevention messages for parents for future outreach, a convenience sample of participants were purposefully recruited from two US states where the lead researcher was located to include a variety of representative and experienced providers. An initial list of healthcare and education providers was developed with input from the project advisory committee and expert input from the authors. Recommendations were based on types and specific providers who have a vested stake and expertise in childhood nutrition and/or obesity prevention. Providers were then contacted by the research team to determine interest in participating in the study. Criteria for participants included being a healthcare or education provider known in the field with a minimum of 5 years work experience, at least 18 years of age, able to speak and read English, and working with families with children between birth to 2 years of age. Participants were recruited in-person, via phone calls, or email. All participants contacted for an interview agreed.

### Interview

To parallel with interviews with parents as part of a simultaneous project [26], the interview questions for this study were based on personal beliefs and behaviors grounded in the Theory of Planned Behavior and the interpersonal factors and dynamic interactions of the Social Cognitive Theory [27]. Questions were reviewed by the study team and project advisory committee for face validity. A trained researcher conducted 60-min interviews with participants individually over the phone, and audio recorded with permission. Questions (Table 1) aimed to determine observed feeding and obesogenic behaviors of families with young children, feeding, physical activity, and screen time, barriers faced in implementing obesity prevention practices, current resource gaps in nutrition education, and dissemination methods. Each provider received a small incentive valued at $10 for his or her time.

**Table 1:** Interview questions with providers to determine perceived practices, barriers and educational opportunities to prevent early childhood obesity of children ages birth to 2 years old

Research Objective	Interview Questions
To determine what feeding practices and obesogenic behaviors of families with 0–2 year olds are observed by providers.	• What are some issues that parents face regarding breastfeeding versus formula feeding? • What are some issues parents face with complementary foods? • What foods or food related practices do parents have the most trouble implementing?
To determine what barriers providers face when implementing early childhood obesity prevention practices with families of young children.	• What are some common misconceptions/confusion that parents have regarding feeding their baby/child? • If parents have mentioned misconceptions these to you, where do these misconceptions tend to come from? • What are the most common reasons why parents do not or are unable to follow the feeding advice or suggestions given? • What reasons do they give for not following it [suggestions for food/drinks parents should avoid giving their children]?
To determine what nutrition education resource gaps providers identify for use with families of 0–2 year olds.	• What other resources would be useful for you (as a provider or within your organization) to further educate parents about feeding their child?

### Data analysis

After the interviews were completed, the audio files were transcribed verbatim and verified by the researchers. Professional titles of providers were used to identify participants within transcripts. All interview questions addressing the research objectives were included in data analysis (Table 1).

Each transcript was analyzed using an open-coding inductive analysis process and analyzed for themes using the classic analysis approach and NVivo Pro 11 [28]. Thematic analysis was then used by a three-member study team to determine key emerging response themes for each question amongst the transcripts [29, 30]. Members of the study team included the lead author (RLH) who was involved in conducting interviews and able to confirm study findings along with two other study authors (ARM, NT) who were not directly involved in conducting interviews but who were able to provide objective analysis to reduce any potential bias. Question response themes were coded and summarized for frequency among interviews by each individual team member independently. Once all transcripts were analyzed by each team member, the study team convened to discuss common findings and confer major response themes. Multiple reviews were used at each stage of qualitative analysis to increase validity of the findings [31].

## Results

The final sample for this study consisted of 21 providers, including early education providers (*n* = 7), community-based providers (*n* = 7), and healthcare providers (*n* = 7). The final sample size was determined once representatives from a variety of professions and roles within the community were included and data saturation was reached based on no further new information provided during the interviews. Emerging themes were summarized for observed feeding practices, barriers to childhood obesity prevention, and nutrition education resource gaps (Table 2).

**Table 2:** Emerging qualitative research themes identified by providers regarding observed feeding practices of parents, barriers and educational opportunities for early childhood obesity prevention

Research Topic	Emerging Themes
Observed Feeding Practices	• Overfeeding in absence of hunger • Early initiation of solids & suboptimal feeding practices • Lack of child autonomy & self-regulation • Suboptimal dietary pattern (Limited vegetable intake, excess high energy-dense food intake)
Barriers to Childhood Obesity Prevention	• Convenience (time and energy for busy parents) • Marketing from social media & food companies • Cultural & familial influences • Lack of knowledge & misconception about healthy foods
Nutrition Education Resource Gaps	• Reputable, evidence-based & accessible information • Culturally/literacy sensitive materials & engaging for parents • Limited resources on transitioning to solid foods (month-specific)

### Observed feeding practices

Overfeeding as it relates to improper portion sizes was one theme that emerged as an observed feeding practice in families with young children. One provider stated, “*They overfeed their kids. They’re not aware of just how small the portion sizes are for kids especially zero to two,”* (Lactation Consultant). A community education observed that, “*Sometimes families think they should be getting a lot more than they actually need, so we actually have a large amount of kids that are actually on the higher end of weight,”* (Early Head Start Education Manager). Providers also reported that families are concerned about their child not receiving enough food, especially during birth to 2 years when their diet is transitioning. One provider explained, “[…] *most commonly, I see overfeeding and parental anxiety about underfeeding. Even though most children are overfed, most parents are worried that they’re underfed,”* (Pediatrician 1). Another provider (Pediatrician 3) indicated that while some parents struggle to have the means to provide enough food, others use food to soothe their child. Parents may experience anxiety and seek other sources to add calories and nutrition into their child’s diet as indicated by a WIC Nutrition Director, *“When the children turn a year old, there’s a significant number of parents […] who are anxious about their child eating well, transitioning off of formula onto food, and so they want a supplemental beverage.”*

Early initiation of solids and inappropriate techniques transitioning to solid food was also an emerging theme. One provider explained *“[…] there’s a misconception about when to give the solid foods or try them. I think a lot of them [parents] are doing it sooner then it’s really recommended.*” “*[…] some of the older [health professionals] are the ones that are encouraging them to start solids sooner than recommendation of around six months.”* (Lactation Consultant). This early initiation was observed as a disregard for current recommendations and was further described as part of a larger phenomenon. One provider explained that parents were *“[…] giving cereal at an earlier age than we recommend because they don’t understand the anatomical needs for the baby to be able to take it safely.*” (Pediatric APRN). This observation included not only a general lack of understanding by parents about when to initiate foods for their infant, but a desire to include the child in family meals. Another provider reported *“[…] they like to try and feed their kids earlier than we would even recommend and not wait – you know how you’re supposed to wait like three days for every food introduced, so that way you see if there’s allergies.*” This became an issue as the provider further explained instances where “*you might have to stop all foods right now, because there’s something going on and because we’ve introduced so many things, we don’t know what it is,*” (Early Head Start Education Director). Another inappropriate technique observed related to early initiation of solid food was introducing solid foods in a baby bottle. One provider described this practice by quoting a parent’s statement of, “*Well, this is what my mom told me to do.’ Cereal in the bottle and the intention really is to make sure they sleep through the night.*” (Lactation Consultant). Another healthcare provider (Pediatrician 3) indicated that colleagues have sometimes suggested adding foods such as cereal to a bottle when a child is struggling to gain weight although it is not recommended practice.

Another feeding practice observed by providers, especially childcare directors, was the lack of autonomy and self-regulation for children ages birth to 2 years during mealtimes. One provider reported that *“[…] one of the biggest challenges is parents are afraid to start little pieces of solid food because they’re afraid their children are going to choke and so we have a lot of challenges with encouraging families to start finger foods,*” (Childcare Director 1). Another provider explained lack of autonomy during mealtime as *“[…] it’s the parent’s worry of choking… and helping the parents to distinguish the difference between the child feeling out a new texture in their mouth and the child actually choking,*” (Childcare Director 3).

The lack of autonomy and self-regulation was also seen with children who were simply not accustomed to feeding themselves. Self-regulation, as it relates to child feeding, is a child’s ability to start and stop eating in response to hunger and fullness cues respectively. A provider explained that “*The parents are always feeding them or they – and so you get these kids coming to us maybe even as toddlers who will have a plate full of food in front of them and they won’t pick it up and put it in their mouth themselves even though physically they’re able to pick up food and put it in their mouth,*” (Childcare Director 1). Another provider indicated*, “The children are not using cups and forks or spoons when they should be. We try to help them transition but it’s not happening at home.”* (Childcare Center Teacher 1) Not only were parents feeding their child, but they also opted to feed their child over letting their child attempt self-feeding. One provider stated, “*So that’s one hard thing for some parents, because it’s easier, quicker, sometimes less messy to feed them themselves, rather than have the child feed themselves,*” (Childcare Director 2). Another source of this feeding behavior was the fear of messy eating. A provider gave details on the response to one parent’s concern, *“The other thing is […], they’re going to make a mess.”* (Childcare Director 1). Concerns of a child making a mess also extended to feeding practices for toddlers at childcare centers where family style serving options were not implemented. (Childcare Center Teacher 2).

The final emerging theme was that of suboptimal dietary patterns during the transition to solid foods. Providers observed that children have too little produce, particularly vegetables, in their diet, and excess high energy-dense foods and added sugars. Some providers (Pediatrician 4 and Registered Dietitian 2) indicated a reduction in diet quality once a child turned 1 year of age or began eating table foods with their family. When asked about foods lacking in the diet during this time, providers clearly stated “*Vegetables. [Their] vegetables and – yeah, vegetables, it’s mostly the children – especially the green vegetables.*” (Community Educator). Although fruit was another concern, providers mentioned that it was variety that was lacking. One provider explained that it was often a financial barrier in regard to increasing fruit variety in the diet. This provider said *“[…] if they buy bananas, they cannot buy apples. If they have the banana and apples, they cannot have the peaches and the plums […]*” (HHC Community Health Educator).

This lack of produce in the diet was not an isolated issue. A provider elaborated by saying, “*In general, […] they don’t have much in the way of fruits and vegetables. They eat excessive amounts of refined carbohydrates and foods that have added sugar […],*” (Pediatrician 2). Some specific examples included “*Lots and lots of juice. And drinks that they perceive as juice like Kool-Aid, lemonade, iced tea. […] They give too much junk food, take-out food, chicken fingers, macaroni and cheese, crackers*,” (Pediatric APRN). There was further concern on sugar-sweetened products such as “*Juice for sure. And sweet stuff. Not enough fruit, but getting their sweet stuff from other candy or cookies.*” (Early Head Start Home Visitor). Another observation by providers was related to foods marketed to parents for children. A provider explained that “*I think parents feel that they need to give their children special foods, baby foods, foods marketed to toddlers […],*” (Pediatric RD 1).

### Barriers to following childhood obesity prevention recommendations

Convenience, such as time and energy needed to prepare food, appeared as one thematic barrier for parents to follow childhood obesity prevention recommendations. One provider explained that some parents do not follow recommendations because “*In the morning sometimes is hard because they’ll do a run to [a fast food donut shop] and then – or they’ll grab something quick […],*” (Childcare Director 2). Besides saving on time, convenience also meant easier choices. Another provider described how parents make less optimal feeding choices simply to avoid conflict, stating, *“I think it’s just easier. They say like their kids are crying and they want it, so they just give it to them,*” (Early Head Start Education Manager). Providers also reported concern that the desire for convenient options led to relying on supplemental products, *“I felt like the whole reason for the toddler formula was just because again it was easier and it was putting this parent’s mind at ease […],*” (Childcare Director 1).

Another observed barrier was the marketing of less healthy or unnecessary foods for young children by social media and food companies. One provider described this occurrence, saying, “*Well, maybe it’s what they’re watching on TV or the advertising on TV.*” (Lactation Consultant). This marketing presence had a clear impact on what parents provide for their children. Another provider observed that “*They want to give [liquid nutrition supplement], or they want to give toddler formula, because advertising is out here,*” (WIC Nutrition Director). The marketing not only influenced parents’ decisions, but created confusion. When asked where misconceptions on child feeding came from, a provider stated “*I think the media is one thing and family and friends. […] and of course now with the internet and chat rooms and all of this, it’s a lot of electronic information,*” (WIC Nutrition Director).

Cultural and familial related influences were another potential barrier to childhood obesity prevention and were a constant obstacle that many providers observed when working with parents. One provider cited an example related to mealtime barriers “*[…] I think that there’s a lot of family input, extended family input so that can be a very hard barrier to break, those cultural norms,*” (Pediatric RD 1). One specific example of cultural influences from the family was that “*if there is an older family member, like, a grandmother who for cultural reasons believes otherwise and […], this last year I had somebody from India, and she said in India they have a special ritual at five months old with the introduction of baby food. And that is their cultural norm and so I just said well, this is what we advise in the United States based upon the AAP recommendation,*” (WIC Nutrition Director). Another example was provided when the provider further stated, “*I think they just go on to the internet or talk with family and friends, and we do have a large Latino population […], and so the Latino culture has some beliefs, early introduction of baby food, putting cereal in the bottle has been one of them,*” (WIC Nutrition Director).

All of these barriers were further exacerbated by the final emerging theme of the lack of knowledge and misconceptions of healthy foods. Providers believed that parents are genuinely concerned about their child receiving proper nutrition, but that parents do not understand what that entails exactly. One provider explained, “*Most people are interested in nutrition. They want to feed their kids healthy foods. They just don’t necessarily know what is healthy and/or unhealthy,*” (Pediatrician 1). On a simpler level, providers observed that some parents see all food as adequate sources of nourishment. This provider elaborated *“[…] for many parents, in a sense, all food is good including commercial food. And so there’s a trust in that and I think sometimes that trust is violated commercially,*” (Pediatrician 2). Even for parents who may be able to differentiate between healthy and unhealthy foods, providers reported there are still misconceptions preventing parents from making healthier choices. The most common of these being that all healthy foods are too expensive to include in their diets. One provider expressed how frequently this issue came up by explaining, “*We hear a lot that it’s expensive. It’s all on the outside of the store we all know. It’s easier to buy a box of hamburger helper. So, there’s a misconception too, eating healthy doesn’t have to cost you a lot of money,*” (Lactation Consultant).

### Nutrition education resource gaps

Community-based and early education providers reported many resource gaps that prevent optimal nutrition education. One main concern was the lack of reputable, evidence-based materials that are accessible to the providers and parents they work with. One provider confirmed, “*I think it would be great for us to have a suggested list of websites that would be considered really valid, approved resources, recommended resources […]*” (Childcare Director 1). Many providers, particularly those working in community-based areas, expressed concerns with accessing these resources, “*If I think it’s a good resource, I find it online and I think it’s a good resource, I’ll give it out. [..] Some of the stuff I really have to put together because I weed through it.*” (Early Head Start Home Visitor).

In addition to reliable materials, community-based and early education providers were also lacking access to nutrition education resources that were sensitive to cultural and literacy differences, and engaging for parents. Providers reported serving families from a variety of cultural backgrounds, and this affected their diet and ability to understand educational materials. One provider explained the challenge “*It’s always been – especially because of the language. We don’t have anything in Spanish, so we have to – I have to do a lot of translation.*” (Community Educator). There was also a lack of nutrition education resources available for low-literacy parents. A provider reported that “*They do have some stuff that we can get out to families, but it’s a lot of reading and the demographic that we serve is lower income and a lot of our families can’t even read past eighth grade level,*” (Early Head Start Education Manager). Further, another provider indicated, *“Parents often lose handouts and it would be good to have something with good photos to show them online or their phone that they can read when they need it.”* (Childcare Center Teacher 2).

Aside from these cultural and literacy needs, healthcare providers identified a lack of engaging resources that can be used with all parents. They also observed that parents did not learn best with current formats of nutrition education materials. Providers explained that parents needed more visual-based resources to capture their attention and help them understand the information: “*I think that what would be most helpful for parents is things that are very visual. So I think we have a lot of information that’s written and I think that’s great, but a lot of the time parents aren’t interested in reading handouts or they’re just not that type of learner and so seeing something that’s even more hands-on or a video or something that’s very visual for them is gonna [sic] be more beneficial,*” (Pediatric RD 1). Another provider indicated, “*An app for their [mobile] phone would be a good way for them [parents] to have information at their fingertips*.” (Pediatrician 3).

In terms of specific topics that were lacking in current nutrition education materials, providers reported that parents needed more information about transitioning infants to solid foods. One provider explained, “*So I think there’s lack of education in that area […] transfer from breastfeeding to regular, table food.*” (Community Educator). Another provider insisted, “*I would love to have feeding for the babies on a monthly basis, […], and up to five years old,*” (HHC Community Health Educator). A provider explained that *“[…] from infant to 2 years old, I also believe that we also need more educational materials […] I have to do a lot of cut and paste because we don’t have a lot of resources.*” (Community Educator).

## Discussion

The findings from this qualitative study identified a provider perspective on early child feeding practices of parents and current education gaps. Overarching themes were categorized as observed feeding practices, barriers to childhood obesity prevention, and nutrition education resource gaps. Within observed feeding practices, providers reported parental overfeeding of young children due to a lack of knowledge and misconception of healthy portion sizes and nutritional requirements for young children. The overall findings of providers’ perceptions intersect and parallel actual concerns and needs of parents as reported in prior research [26]. Previous studies found that mothers from diverse populations primarily associated crying and distress with hunger [32, 33] and the belief that infants should finish the entire bottle at feeding times, increasing obesity risk [32–34]. Parents also often serve inappropriate portions linked to increased energy intake [35, 36].

Early introduction of solid foods by parents was also of concern to providers as it relates to observed feeding practices and was associated with a lack of knowledge and cultural or familial influences. Although some research indicates that parents are aware of the importance to delay solid foods [37], previous research found that early introduction of complementary foods was associated with diets high in energy dense snacks and sweets and lower in fruits and vegetables [38, 39]. Additional studies have shown that early introduction of solid foods was positively associated with obesity outcomes at 3 and 6 years of age [40, 41]. In these studies, early introduction of solid foods was associated with lower maternal education and racial and ethnic differences, as Black and Hispanic mothers were more likely to implement early introduction of solid foods [42].

The lack of autonomy and self-regulation was associated with parents’ safety concerns. Lower self-regulation is associated with lower parental control during feeding [43], increased weight status, and lower executive function related to eating in children [43, 44]. Providers reported parents concern about child’s safety and messiness during self-feeding. This may be due to limited knowledge and confidence on how to identify and react to choking hazards. There is also the possible lack of understanding for the greater benefits from self-feeding [45, 46].

When examining other barriers to childhood obesity prevention practices by parents as reported by providers, convenience such as time and energy for busy parents to implement optimal feeding practices was identified as a key barrier. This is consistent with prior research that has been previously identified time as a barrier to healthy feeding practices by parents [47]. Providers also identified marketing of foods to parents of infants and toddlers as a barrier. Accordingly, prior research has indicated that parents and caregivers may be misled by food marketing messages to purchase less optimal infant formula and toddler milks [48].

Providers also identified nutrition education resource gaps. Although medical healthcare providers reported access to more reliable resources, early education and community-based providers reported limited access to evidence-based educational materials. Providers, especially in healthcare settings, indicated the need for more engaging, culturally appropriate, visually appealing, interactive materials at appropriate literacy levels. Consequently, another study identified that low parent health literacy significantly increased the use of obesogenic child feeding practices [49]. Providers also expressed the need for materials translated in Spanish and to include culturally appropriate foods and practices as also recommended by other educators and researchers [50].

The strengths of the study include that it was a timely, in-depth qualitative analysis of a variety of providers’ opinions about misconceptions and barriers that prevent parents from implementing early childhood feeding and obesity prevention practices. While the results cannot be generalized to all geographic areas, providers were recruited from two US states. The limitations of this study include a small sample size although saturation was reached. While the sample included providers from a variety of specialties and serving diverse populations from two states as recommended by the project advisory committee and study authors, there may still be other health or education professionals or opinions that were not represented.

## Conclusion

It is clear that misperceptions and conflicting information about early childhood feeding continue to impact parents of young children. Providers are in need of more or better educational resources based on these considerations especially considering that dietary guidance has been recently been included for children ages 0 to 2 years old in the US Dietary Guidelines for Americans for the first time in history. Early education and community-based providers reported limited access to evidence-based educational materials more so than healthcare providers did. Providers, particularly in healthcare settings, indicated the need for more engaging, culturally appropriate, visually appealing, interactive materials at appropriate literacy levels. Consequently, lower health literacy in parents has been associated with significantly increased use of obesogenic child feeding practices [49]. Providers also expressed the need for materials in Spanish and to include culturally appropriate foods and practices, characteristics of educational materials deemed important in health promotion including for parents of toddlers [50, 51].

Overall, based on the results, there is a need for readily available, evidence-based, engaging patient education materials for provider interactions with parents of infants and toddlers to address all transitions of early childhood feeding. Further research is needed to evaluate what specific content as it relates to early childhood feeding and dissemination methods such as handouts, videos, or digital technology are most effective in preventing early childhood obesity when distributed by providers to parents of infants and toddlers.

## Data Availability

The thematic data and materials used in this study are available from the corresponding author upon reasonable request.

## References

[1] National Institutes of Health and National Heart Lung and Blood Institute (2017). Overweight and Obesity.

[2] Centers for Disease Control and Prevention (2017). Childhood obesity facts.

[3] Hales CM (2017). Prevalence of Obesity Among Adults and Youth: United States, 2015-2016. NCHS Data Brief.

[4] Ogden CL (2015). Prevalence of Obesity Among Adults and Youth: United States, 2011-2014. NCHS Data Brief.

[5] Siega-Riz AM, Deming DM, Reidy KC, Fox MK, Condon E, Briefel RR (2010). Food consumption patterns of infants and toddlers: where are we now?. J Am Diet Assoc.

[6] Birch LL, Doub AE (2014). Learning to eat: birth to age 2 y. Am J Clin Nutr.

[7] Hoffmann DA, Marx JM, Kiefner-Burmeister A, Musher-Eizenman DR (2016). Influence of maternal feeding goals and practices on children's eating behaviors. Appetite..

[8] Kiefner-Burmeister AE, Hoffmann DA, Meers MR, Koball AM, Musher-Eizenman DR (2014). Food consumption by young children: a function of parental feeding goals and practices. Appetite..

[9] Patrick H, Nicklas TA (2005). A review of family and social determinants of children's eating patterns and diet quality. J Am Coll Nutr.

[10] Birch LL, Davison KK (2001). Family environmental factors influencing the developing behavioral controls of food intake and childhood overweight. Pediatr Clin N Am.

[11] Rhee K (2008). Childhood overweight and the relationship between parent behaviors, parenting style, and family functioning. ANNALS Am Acad Polit Soc Sci.

[12] Kroller K, Warschburger P (2008). Associations between maternal feeding style and food intake of children with a higher risk for overweight. Appetite..

[13] Savage JS, Birch LL, Marini M, Anzman-Frasca S, Paul IM (2016). Effect of the INSIGHT responsive parenting intervention on rapid infant weight gain and overweight status at age 1 year: a randomized clinical trial. JAMA Pediatr.

[14] Wen LM (2012). Effectiveness of home based early intervention on children’s BMI at age 2: randomised controlled trial. Bmj.

[15] Hughes SO, Patrick H, Power TG, Fisher JO, Anderson CB, Nicklas TA (2007). The impact of child care providers’ feeding on children’s food consumption. J Dev Behav Pediatr.

[16] Kim J, Peterson KE (2008). Association of infant child care with infant feeding practices and weight gain among US infants. Arch Pediatr Adolesc Med.

[17] Matvienko-Sikar K, Toomey E, Delaney L, Harrington J, Byrne M, Kearney PM, Choosing Healthy Eating for Infant Health (CHErIsH) study team (2018). Effects of healthcare professional delivered early feeding interventions on feeding practices and dietary intake: a systematic review. Appetite..

[18] Spiby H, McCormick F, Wallace L, Renfrew MJ, D’Souza L, Dyson L (2009). A systematic review of education and evidence-based practice interventions with health professionals and breast feeding counsellors on duration of breast feeding. Midwifery..

[19] Gartner LM, Morton J, Lawrence RA, Naylor AJ, O'Hare D, Schanler RJ, Eidelman AI, American Academy of Pediatrics Section on Breastfeeding (2005). Breastfeeding and the use of human milk. Pediatrics..

[20] Gidding SS, Dennison BA, Birch LL, Daniels SR, Gillman MW, Lichtenstein AH, Rattay KT, Steinberger J, Stettler N, van Horn L, American Heart Association, American Academy of Pediatrics (2005). Dietary recommendations for children and adolescents: a guide for practitioners: consensus statement from the American Heart Association. Circulation..

[21] American Academy of Pediatrics, K., R. E., & Greer, F. R. . Pediatric nutrition : policy of the American Academy of Pediatrics. American Academy of Pediatrics. 2013.

[22] Dwyer JT, Butte NF, Deming DM, Siega-Riz AM, Reidy KC (2010). Feeding infants and toddlers study 2008: progress, continuing concerns, and implications. J Am Diet Assoc.

[23] Ciampa PJ, Kumar D, Barkin SL, Sanders LM, Yin HS, Perrin EM, Rothman RL (2010). Interventions aimed at decreasing obesity in children younger than 2 years: a systematic review. Arch Pediatr Adolesc Med.

[24] US Department of Agriculture and US Department of Health and Human Services (2020). Dietary Guidelines for Americans.

[25] Matvienko-Sikar K, Kelly C, Sinnott C, McSharry J, Houghton C, Heary C, Toomey E, Byrne M, Kearney PM (2018). Parental experiences and perceptions of infant complementary feeding: a qualitative evidence synthesis. Obes Rev.

[26] Heller RL, Chiero JD, Puglisi M, Mobley AR (2019). Feeding infants and toddlers: a qualitative study to determine parental education needs. Child Obes.

[27] Bandura A (2004). Health promotion by social cognitive means. Health Educ Behav.

[28] Thomas DR (2006). A general inductive approach for analyzing qualitative evaluation data. Am J Eval.

[29] Braun V, Clarke V (2006). Using thematic analysis in psychology. Qual Res Psychol.

[30] Tuckett AG (2005). Applying thematic analysis theory to practice: a researcher's experience. Contemp Nurse.

[31] Patton MQ (1999). Enhancing the quality and credibility of qualitative analysis. Health Serv Res.

[32] Gross RS, Fierman AH, Mendelsohn AL, Chiasson MA, Rosenberg TJ, Scheinmann R, Messito MJ (2010). Maternal perceptions of infant hunger, satiety, and pressuring feeding styles in an urban Latina WIC population. Acad Pediatr.

[33] Redsell SA, Atkinson P, Nathan D, Siriwardena AN, Swift JA, Glazebrook C (2010). Parents’ beliefs about appropriate infant size, growth and feeding behaviour: implications for the prevention of childhood obesity. BMC Public Health.

[34] Worobey J, Peña J, Ramos I, Espinosa C (2014). Infant difficulty and early weight gain: does fussing promote overfeeding?. Matern Child Nutr.

[35] Fisher JO, Liu Y, Birch LL, Rolls BJ (2007). Effects of portion size and energy density on young children's intake at a meal. Am J Clin Nutr.

[36] Johnson SL, Hughes SO, Cui X, Li X, Allison DB, Liu Y, Goodell LS, Nicklas T, Power TG, Vollrath K (2014). Portion sizes for children are predicted by parental characteristics and the amounts parents serve themselves. Am J Clin Nutr.

[37] Gijsbers B, Mesters I, André Knottnerus J, Legtenberg AHG, van Schayck CP (2005). Factors influencing breastfeeding practices and postponement of solid food to prevent allergic disease in high-risk children: results from an explorative study. Patient Educ Couns.

[38] Grummer-Strawn LM, Scanlon KS, Fein SB (2008). Infant feeding and feeding transitions during the first year of life. Pediatrics..

[39] Santos LP, Assunção MCF, Matijasevich A, Santos IS, Barros AJD (2016). Dietary intake patterns of children aged 6 years and their association with socioeconomic and demographic characteristics, early feeding practices and body mass index. BMC Public Health.

[40] Huh SY, Rifas-Shiman SL, Taveras EM, Oken E, Gillman MW (2011). Timing of solid food introduction and risk of obesity in preschool-aged children. Pediatrics..

[41] Imai CM (2014). Associations between infant feeding practice prior to six months and body mass index at six years of age. Nutrients..

[42] Taveras EM, Gillman MW, Kleinman KP, Rich-Edwards JW, Rifas-Shiman SL (2013). Reducing racial/ethnic disparities in childhood obesity: the role of early life risk factors. JAMA Pediatr.

[43] Hughes SO, Frazier-Wood AC (2016). Satiety and the self-regulation of food take in children: a potential role for gene-environment interplay. Curr Obes Rep.

[44] Posne MI, Rothbart MK (2000). Developing mechanisms of self-regulation. Dev Psychopathol.

[45] Tan CC, Holub SC (2011). Children's self-regulation in eating: associations with inhibitory control and parents’ feeding behavior. J Pediatr Psychol.

[46] Wehrly SE, Bonilla C, Perez M, Liew J (2014). Controlling parental feeding practices and child body composition in ethnically and economically diverse preschool children. Appetite..

[47] Martin-Biggers J, Spaccarotella K, Hongu N, Alleman G, Worobey J, Byrd-Bredbenner C (2015). Translating it into real life: a qualitative study of the cognitions, barriers and supports for key obesogenic behaviors of parents of preschoolers. BMC Public Health.

[48] Romo-Palafox MJ, Pomeranz JL, Harris JL (2020). Infant formula and toddler milk marketing and caregiver's provision to young children. Matern Child Nutr.

[49] Yin HS, Sanders LM, Rothman RL, Shustak R, Eden SK, Shintani A, Cerra ME, Cruzatte EF, Perrin EM (2014). Parent health literacy and “obesogenic” feeding and physical activity-related infant care behaviors. J Pediatr.

[50] Kreuter MW, Lukwago SN, Bucholtz DC, Clark EM, Sanders-Thompson V (2003). Achieving cultural appropriateness in health promotion programs: targeted and tailored approaches. Health Educ Behav.

[51] Horodynski MA, Brophy-Herb H, Henry M, Smith KA, Weatherspoon L (2009). Toddler feeding: expectations and experiences of low-income African American mothers. Health Educ J.

